# Soft tissue inflammation of the lower limb and foot following tibiotalocalcaneal arthrodesis for osteoarthritis in a morbidly obese patient: A case report

**DOI:** 10.1097/MD.0000000000046903

**Published:** 2026-01-09

**Authors:** Kamil Dworak, Jarosław Pasek, Sonia Rokicka, Krystian Werner, Maciej Szczęśniak, Maciej Tarski, Artur Mikuszewski, Tomasz Stołtny

**Affiliations:** a Department of Orthopedics, District Hospital of Orthopedics and Trauma Surgery in Piekary Śląskie, Piekary Śląskie, Poland; b Faculty of Medical Sciences, Collegium Medicum, Jan Długosz University in Częstochowa, Częstochowa, Poland; c Faculty of Medicine, Medical University of Gdańsk, Gdańsk, Poland.

**Keywords:** foot, inflammation, lower leg, soft tissue, surgical treatment, tibiotalocalcaneal arthrodesis

## Abstract

**Rationale:**

Tibiotalocalcaneal arthrodesis (TTCA) is a radical orthopedic procedure performed in patients with severe ankle and subtalar joint deformities, loss of limb support function, and chronic pain. TTCA in obese patients with multiple comorbidities carries a particularly high risk of postoperative complications. Reporting such cases helps highlight potential challenges and guide clinical management.

**Patient concerns:**

We present a case of a 64-year-old morbidly obese patient (BMI 49.3 kg/m²) with a history of bariatric surgery who underwent TTCA.

**Diagnoses:**

Surgical site infection is a common early (around 3%) and late postoperative complication (up to 60%) following TTCA.

**Interventions:**

The patient was treated with targeted antibiotic therapy, surgical debridement with resection of necrotic tissue, and placement of a Tiersch graft, which fully healed within 7 weeks.

**Outcomes:**

Nine months after surgery, the patient developed soft tissue inflammation in the lower limb and foot as a late complication associated with the intramedullary heel nail used during TTCA.

**Lessons:**

Combined surgical and antibiotic treatment allowed complete healing and return to full physical activity. This case emphasizes that pantalar arthrodesis in obese patients with multimorbidity carries a significantly higher risk of surgical site infection and requires careful perioperative management.

## 1. Introduction

Tibiotalocalcaneal arthrodesis (TTCA) is a commonly used surgical method for stiffening the ankle and subtalar joints in cases of advanced degenerative changes.^[1]^ The primary goals of this procedure are to reduce pain, restore the proper alignment of the lower leg, and improve the limb’s weight-bearing function. Other arthrodesis techniques for the tibiotalar and subtalar joints include open methods such as the Adams and Campbell procedures, as well as combined approaches. The advantage of TTCA lies in its minimally invasive nature, as it avoids previously performed surgical incisions by employing a “closed” technique.^[1,2]^ Despite its limited surgical exposure, TTCA is associated with a range of complications, occurring in approximately 2% to 15% of cases. Superficial infections occur in 3% to 8% of patients, while deep infections affect 1% to 7%.^[1,3]^ The most commonly reported complications include limb shortening, persistent surgical site infection (SSI), nonunion of the bone, and ongoing pain in the operated area. Among the independent factors influencing the success of the surgical treatment, besides the surgeon’s expertise, are reduced bone density due to osteoporosis and the presence of comorbidities such as diabetes mellitus, rheumatoid arthritis, and atherosclerosis obliterans.^[4–6]^

## 2. Aim of the study

The aim of the study is to present the surgical management and treatment outcomes of a 64-year-old patient who developed soft tissue inflammation of the lower leg and foot as a postoperative complication following tibiotalocalcaneal arthrodesis with intramedullary heel nail fixation.

## 3. Case report

A 64-year-old patient (JH) presented to the trauma and orthopedic clinic in 2022 with a 3-year history of right ankle pain (VAS score 9/10) and progressive functional impairment. The patient had severe obesity (BMI 49.3 kg/m²) and hypertension and had previously undergone bariatric surgery (stomach reduction) in 2021. He mobilized using 2 elbow crutches and exhibited a limp on the right lower limb.

Imaging studies, including X-ray and CT scans of the ankle joint, along with physical examination, indicated the need for surgical intervention. In August 2022, the patient underwent tibiotalocalcaneal arthrodesis (TTCA) of the right ankle using a ChM nail. The procedure and early postoperative course were uneventful.

**Figure 1.**
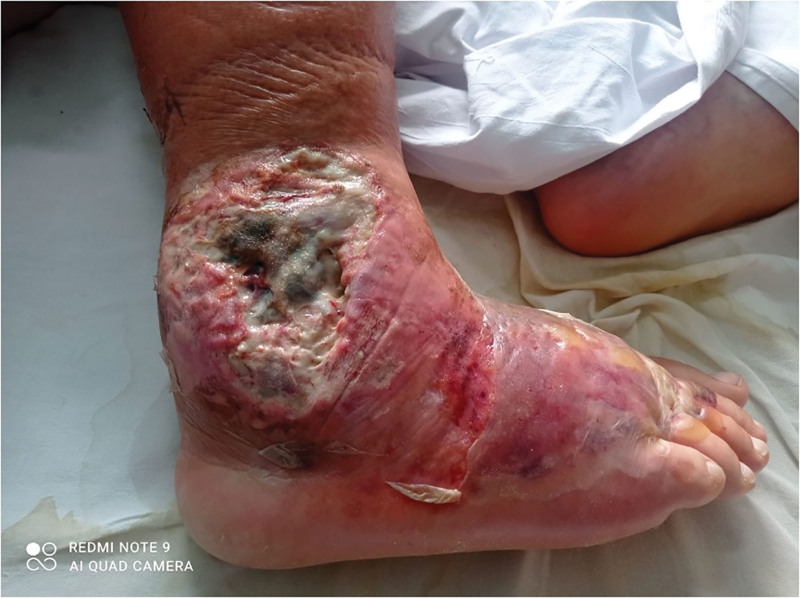
The area above the lateral malleolus of the right lower leg with visible necrotic changes.

**Figure 2.**
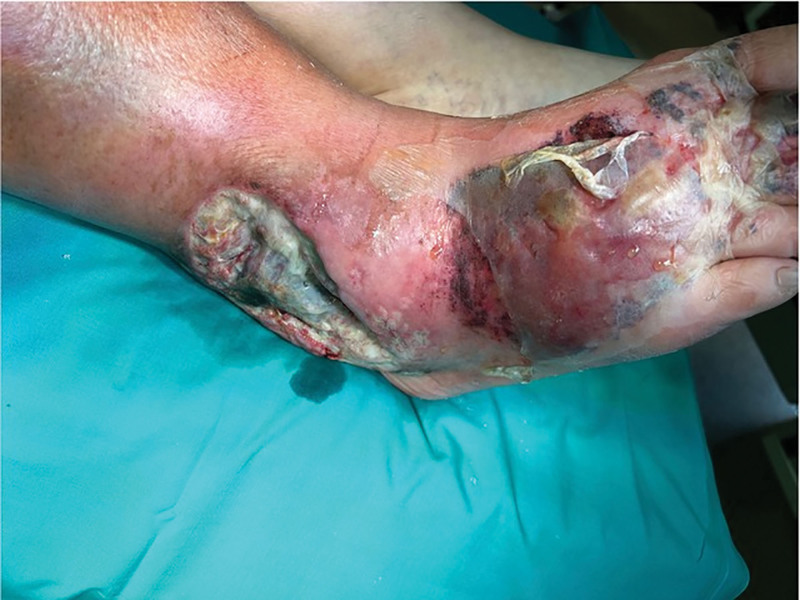
The area above the lateral malleolus of the right lower leg and right foot with visible necrotic changes on the day of hospital admission.

Nine months after surgery, a spontaneous local separation of the postoperative scar (1.5 cm) appeared in the area of the lateral malleolus. Due to worsening local and systemic condition, the patient was admitted to the hospital. An initial swab was negative, and empirical antibiotic therapy was administered for 21 days. Outpatient vascular consultation was performed. Subsequent culture identified Staphylococcus aureus, prompting targeted intravenous antibiotic therapy (Syntarpen 4 × 1 g and Augmentin 2 × 1 g for 21 days) along with anticoagulation (low molecular weight heparin, Clexane 0.04 mL). Despite pharmacological management, extensive soft tissue inflammation developed around the lateral malleolus and dorsum of the right foot (Figs. 1 and 2).

**Figure 3.**
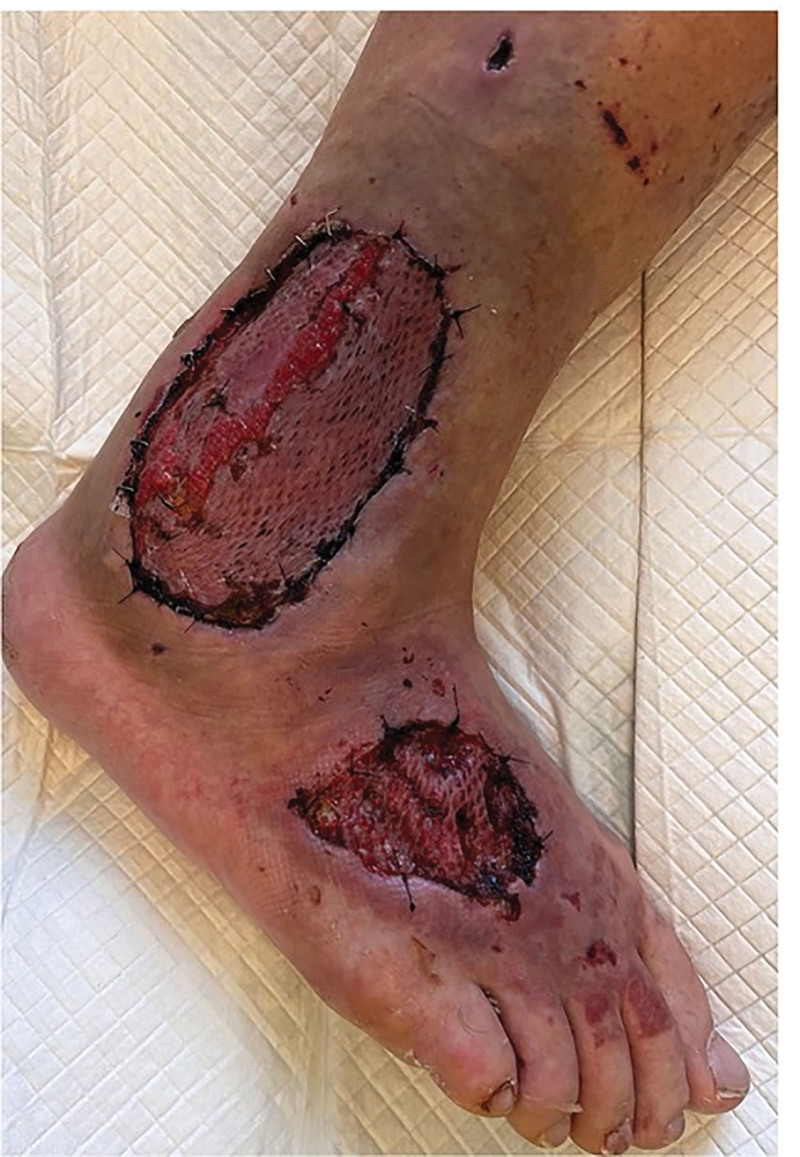
The area above the lateral malleolus of the right lower leg and right foot following the aforementioned procedures.

The patient was subsequently qualified for surgical debridement, including extensive resection of necrotic tissue of the right lower leg and foot. Daily wound care led to granulation. After 2 weeks, 2 Tiersch grafts were performed (one with Mersilene mesh) from the right thigh to the lateral malleolus and dorsum of the right foot (Fig. 3).

**Figure 4.**
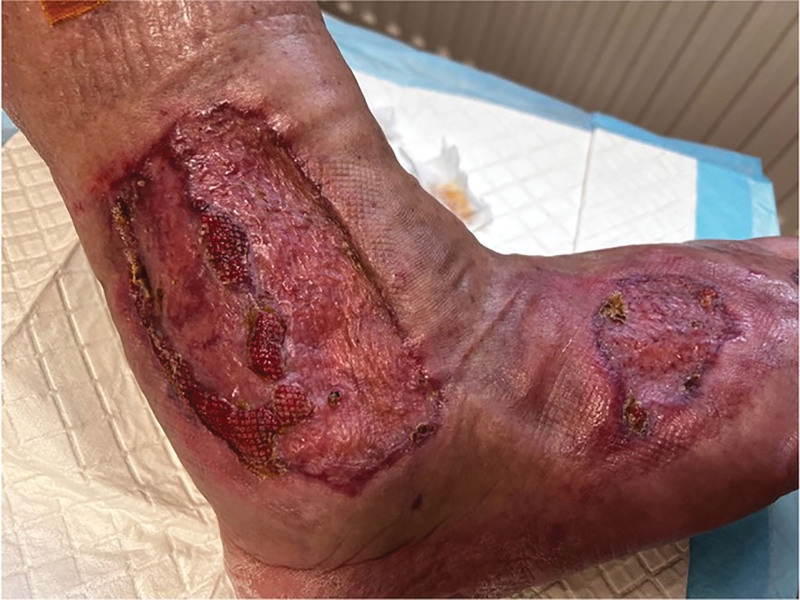
The area above the lateral malleolus of the right lower leg and right foot with visible healing of the Tiersch grafts (on the day of hospital discharge).

**Figure 5.**
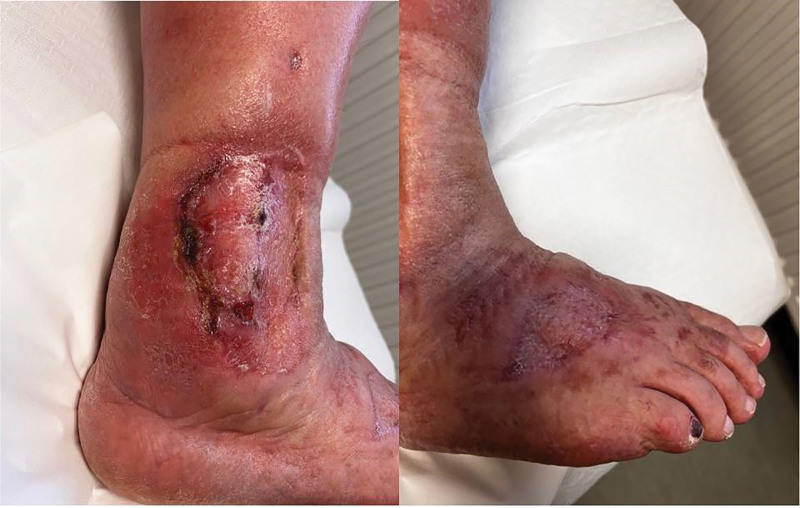
The area above the lateral malleolus of the right lower leg and right foot following completion of treatment.

Postoperatively, the patient remained in the ward for 4 weeks (Fig. 4) and was then monitored at a local orthopedic clinic 3 times weekly under continuous supervision (Fig. 5).

The final stage of treatment involved removal of the implant used for stabilization in September 2024, 2 years after the initial procedure.

## 4. Results

The combined surgical and pharmacological therapy led to complete resolution of soft tissue inflammation and full healing of the grafts within 7 weeks. Pain levels decreased significantly (VAS score reduced from 9 to 2). At final follow-up, the patient was fully mobile without assistive devices, reported no recurrent infection, and had returned to normal daily activities.

## 5. Discussion

The constantly increasing number of orthopedic procedures performed to correct extensive joint deformities in patients with multiple diseases is associated with an increase in the percentage of complications. The above-mentioned correlation is directly related to the aging of society and the need to restore an independent existence.^[7–9]^

In order to reduce the number of postoperative complications in the above-mentioned patients, the patient’s condition should be optimized and interventions should be directed towards potential risk factors. The risk of infection in a patient can be assessed before surgery using screening methods and clear communication between the patient and the physician. The most common modifiable risk factors in 80% of patients qualified for orthopedic procedures are obesity, anemia, malnutrition and decompensated diabetes. Other factors requiring preoperative screening include: rheumatoid arthritis, smoking, excessive alcohol consumption, renal failure, cardiovascular problems and depression.^[1–3,10]^

In this work, we presented the result of combined treatment of a 64-year-old patient with grade III obesity who developed a SSI 9 months after the initial surgical treatment. Due to the growing problem of obesity causing the development of numerous complications in Poland, Europe and the United States, its role as a significant factor increasing the risk of SSI is increasing. In 2019, according to data from the Central Statistical Office, 56.6% of people in Poland over 15 years of age were diagnosed as overweight or obese (of which obesity affected 18.5% of people). According to NCD Risk Factor Collaboration, in Poland in 2025 obesity will affect 25.9% of women and 30.3% of men aged 20 or more.^[11]^

Adipose tissue is poorly vascularized, functions as an endocrine organ releasing numerous proinflammatory cytokines, which promotes infection. Operations in obese patients are technically more difficult and take longer, and rich adipose tissue promotes the formation of dead spaces and hematomas. Finally, it easily necroses, providing an environment conducive to the development of infection. According to Antonelli, the risk of SSI is twice as high in obese patients undergoing orthopedic surgery.^[6]^ In this group of patients, other skin lesions are also often found, and the general bacterial contamination of the skin is higher (especially in the case of concomitant, improperly treated or decompensated diabetes).^[4]^

Due to extensive deformation of the tibiotalar and subtalar joints, the patient was qualified for a radical and definitive surgical procedure. The pantalar fixation procedure was without complications. However, after 9 months, the surgical site became infected, which required the implementation of further therapeutic procedures.

Rana et al (2021) also assessed in their study the role of arthrodesis as a rescue procedure in neuroarthropathic Charcot foot and ankle deformities in diabetic patients due to the relatively high rate of complications described in the literature. The obtained study results showed a very low rate of complications, which indicates very good preparation of patients for arthrodesis procedures. There were only 4 superficial infections and 2 deep infections (13%).^[12]^

Geurra Alvarez et al (2022) retrospectively analyzed 7 patients from 2016 to 2019 who underwent minimally invasive arthroscopic pantalar arthrodesis using a retrograde intramedullary nail. Only 1 patient developed wound complications that required subsequent surgery, and another patient was found to have pseudo-arthritis of the tibiotalar ankle, although asymptomatic.^[13]^

Goebel et al (2006) evaluated 29 patients who underwent pantalar arthrodesis with a retrograde femoral nail inserted via a plantar approach in a prospective study. Inflammatory complications occurred in 6 patients (21%), including 2 deep infections, 3 nonunions, and 1 case of postoperative flexion deformity.^[14]^

Fenton et al (2014) in their retrospective study indicate that serious complications may occur after arthrodesis. 9 of 52 operated patients developed deep infection, and in 6 patients the limb was saved by removing the metal elements, cleaning the wound and implanting cement with antibiotics. The remaining 3 patients underwent below-knee amputation.^[10]^

McKinley et al (2011) evaluated ankle arthrodesis in 62 patients. Postoperative complications included early infection treated with antibiotics and early surgical revision with irrigation in 2 (3.2%) patients with RA who were receiving biologic therapy. Late infection developed 2 to 3 years after surgery in 3 (4.3%) patients (2 had RA). Infection was treated with revision surgery with nail removal and irrigation.^[2]^

Anderson et al (2005) reviewed 25 patients who underwent pantalar arthrodesis with an intramedullary nail in patients with rheumatoid arthritis. The authors noted 3 deep infections, all healed. In 2 cases, after the development of nailfold inflammation, the arthrodesis healed.^[15]^

Li W et al (2024) investigated the effect of single-stage arthroscopic tibiotalar and subtalar arthrodesis (TTCA) with external fixation in the treatment of septic arthritis of the ankle and rearfoot. This retrospective study included 6 patients diagnosed with acute or chronic septic arthritis of the ankle or rearfoot who underwent surgical intervention consisting of thorough debridement, single-stage ankle fixation or arthroscopic-assisted TTCA with external fixation. Bone union was achieved in all patients, and the infection reduction rate was 100%.^[16]^

This case report presents a unique clinical scenario of late SSI occurring 9 months after tibiotalocalcaneal arthrodesis in a patient with severe obesity and multiple comorbidities. To our knowledge, few studies have detailed the combined approach of extensive surgical debridement with Tiersch grafting and targeted antibiotic therapy in such a complex patient profile, highlighting a practical treatment pathway for managing late complications in this high-risk group.^[17,18]^

Moreover, this case highlights the importance of a multidisciplinary and individualized approach in managing postoperative infections in patients with significant comorbidities such as severe obesity. The combined use of targeted antibiotic therapy, surgical debridement, and skin grafting (Tiersch graft) proved effective in resolving the infection and promoting wound healing. These findings align with recent evidence emphasizing the need for tailored treatment protocols to optimize outcomes in complex cases following TTCA.^[19]^

## 6. Limitations of the study

This study reports a single case of a patient with severe obesity, multiple comorbidities, and prior bariatric surgery. Therefore, the findings may have limited generalizability to broader patient populations. Further studies with larger cohorts are needed to confirm the effectiveness of combined surgical and pharmacological management of late SSIs following tibiotalocalcaneal arthrodesis.

## 7. Conclusions

Considering the above case description to determine the cause of the soft tissue inflammation, the first factor to suspect is the initial high BMI, which is combined with poorly vascularized adipose tissue where a hematoma remained after surgical treatment. These 2 factors ultimately caused inflammation and secondary infection, because such skin has a greater affinity for bacterial infection. The combined treatment used allowed for complete healing of the grafts and the patient’s return to full physical activity. Pantalar arthrodesis in patients with obesity and a history of multimorbidity unfortunately carries a higher risk of infection of the surgical site.

## Author contributions

**Conceptualization:** Kamil Dworak, Tomasz Stołtny.

**Data curation:** Kamil Dworak, Sonia Rokicka, Krystian Werner.

**Formal analysis:** Jarosław Pasek, Sonia Rokicka, Krystian Werner, Maciej Szczęśniak, Maciej Tarski, Artur Mikuszewski, Tomasz Stołtny.

**Investigation:** Kamil Dworak, Jarosław Pasek, Maciej Tarski.

**Methodology:** Tomasz Stołtny.

**Resources:** Kamil Dworak, Jarosław Pasek.

**Software:** Kamil Dworak, Jarosław Pasek, Sonia Rokicka, Tomasz Stołtny.

**Supervision:** Jarosław Pasek, Artur Mikuszewski.

**Visualization:** Krystian Werner, Maciej Szczęśniak.

**Writing – original draft:** Kamil Dworak, Jarosław Pasek, Krystian Werner, Maciej Szczęśniak, Maciej Tarski, Artur Mikuszewski.

**Writing – review & editing:** Kamil Dworak, Jarosław Pasek, Sonia Rokicka, Tomasz Stołtny.
